# Analysis of Connectivity in Electromyography Signals to Examine Neural Correlations in the Activation of Lower Leg Muscles for Postural Stability: A Pilot Study

**DOI:** 10.3390/bioengineering12010084

**Published:** 2025-01-17

**Authors:** Gordon Alderink, Diana McCrumb, David Zeitler, Samhita Rhodes

**Affiliations:** 1 Department of Physical Therapy & Athletic Training, Grand Valley State University, Grand Rapids, MI 49503, USA; 2 BAMF Health, Grand Rapids, MI 49503, USA; mccrumbdjs@gmail.com; 3 Department of Statistics, Grand Valley State University, Allendale, MI 49401, USA; zeitlerd@mail.gvsu.edu; 4 School of Engineering, Grand Valley State University, Grand Rapids, MI 49504, USA; rhodesam@gvsu.edu

**Keywords:** postural control, balance, ankle strategy, common neural drive, magnitude-squared coherence

## Abstract

In quiet standing, the central nervous system implements a pre-programmed ankle strategy of postural control to maintain upright balance and stability. This strategy comprises a synchronized common neural drive delivered to synergistically grouped muscles. This study evaluated connectivity between EMG signals of the unilateral and bilateral homologous muscle pairs of the lower legs during various standing balance conditions using magnitude-squared coherence (MSC). The leg muscles examined included the right and left tibialis anterior (TA), medial gastrocnemius (MG), and soleus (S). MSC is a frequency domain measure that quantifies the linear phase relation between two signals and was analyzed in the alpha (8–13 Hz), beta (13–30 Hz), and gamma (30–100 Hz) neural frequency bands for feet together and feet tandem, with eyes open and eyes closed conditions. Results showed that connectivity in the beta and lower and upper gamma bands (30–100 Hz) was influenced by standing balance conditions and was indicative of a neural drive originating from the motor cortex. Instability was evaluated by comparing less stable standing conditions with a baseline—eyes open feet together stance. Changes in connectivity in the beta and gamma bands were found to be most significant in the muscle pairs of the back leg during a tandem stance regardless of dominant foot placement. MSC identified the MG:S muscle pair as significant for the right and left leg. The results of this study provided insight into the neural mechanism of postural control.

## 1. Introduction

The evolution of the human bipedal stance and the ability to maintain standing balance during activities of daily living were simultaneous. The standing posture of humans is characterized by a narrow base of support, a higher location of the center of mass, and a propensity for biomechanical instability. Functional decline with age increases postural sway [1,2]. Also, patients with neurological and musculoskeletal disorders have demonstrated poor balance control [3,4,5,6] and have a high fall risk [7,8,9,10,11]. To prevent falls, the biomechanics and neural control of quiet standing balance in healthy young and elderly adults and individuals with disease, e.g., neuropathology, needs to be clarified.

The central nervous system (CNS) facilitates the integration of visual, vestibular, and somatosensory information to coordinate muscle actions associated with static postures and dynamic movements [12]. The musculoskeletal system comprises approximately 700 muscles crossing over 300 joints. To generate movement, individual muscles are activated via the coordination of thousands of individual motor units (MU) through their motor pools. Thus, achieving specific behavioral goals through muscle action redundancy creates a degree of freedom (DoF) problem [13,14]. Bernstein’s [13] hierarchical control theory suggested that the CNS implemented specific functional control structures to limit the DoF at four levels: muscle tone, muscle synergies, space, and actions [13,15]. The neural mechanisms that organize and control human standing posture and movement have not yet been entirely elucidated.

The simplest definition of muscle synergies (the definition we will assume) is groups of muscles that act together to complete a similar act or function. The notion that muscle synergies are described as functional “structures” that contain the minimal number of muscles needed to generate a movement and accomplish a behavioral goal is a hallmark of neurophysiological approaches to explain coordinated movement. These neurophysiological approaches presume that muscle synergies result in the translation of task-level neural commands into execution-level muscle activation patterns as the “solution” to the DoF problem [16,17]. Latash et al. [16] offered an alternative view of muscle synergies. Thus, if an element, i.e., muscle, introduced an error into the common output, other elements would change their contributions to minimize the original error; systems that function according to that principle and demonstrate error compensation among the elements would be called synergies [18]. This notion of compensatory elements was referred to as the “principle of abundance”, which suggests that all the elements (DoFs) always participate in all the tasks, assuring both the stability and flexibility, i.e., variability, of the performance, and, in essence, rendering the redundancy problem irrelevant [16,17].

In attempts to elucidate the role that muscle synergies might play in addressing the DoF problem, several different approaches have been used. Krishnamoorthy et al. [19,20] used the integrated electromyography (EMG) of several postural muscles and principal component analysis (PCA) to define muscle (or M) modes. Torres-Oviedo and Ting [21] used non-negative matrix factorization (NNMF) to identify muscle synergies related to human postural control to examine the intra- and inter-trial variation, while Boonstra et al. [22] decomposed EMG envelopes from ten leg muscles using NNMF and PCA to estimate the number of muscle synergies during a variety of standing activities. Others have used EMG data as input for event-driven intermittent feedback control [23] or neuromechanical [24] models.

In the last three decades, efforts to understand how the CNS controls synergistic muscle activity have focused on examining the role of common neural drives that synchronously activate motor pools of individual muscles in a functional synergy as a single unit at various frequencies [25,26,27,28,29,30,31]. A common neural drive that simultaneously activates multiple motor pools also synchronizes their firing rate [32]. The frequency ranges associated with neural drive oscillations indicate their signal origin in the CNS [26], as shown in Table 1. The strength of neural synchronization from these frequency bands is identified through connectivity analysis, i.e., EMG-EMG coherence (magnitude-squared coherence). Methods using corticomuscular coherence (CMC) and intermuscular coherence (IMC) have been used to investigate common inputs to groups of muscles related to quiet bipedal standing [33,34], ballet postures [35], squatting [36], heel rise tasks [37], as well as sitting and standing tasks in patients with Parkinson’s disease [38]. After the introduction of several fundamental concepts that immediately follow, the remainder of the review will focus on IMC related to investigations on human balance during quiet bipedal standing.

### 1.1. Human Balance and Postural Control

Human balance refers to a state of equilibrium where the center of pressure (COP) oscillates about the body’s center of mass (COM) that lies within a base of support (BOS) to prevent a fall [39,40,41,42,43,44,45]. Due to a relatively large COM located two-thirds of the body height above ground, humans are inherently unstable bipeds that need a continuously active control system to maintain balance and stability [40,46]. Stability is the inherent ability to achieve, maintain, and/or restore a balanced state to avoid a fall [44]. Posture describes the orientation of the multi-segmental human body relative to gravity [40]. Note that balance and posture are often used interchangeably and in combination to assess and define the stability/instability of normal healthy humans.

**Figure 1:**
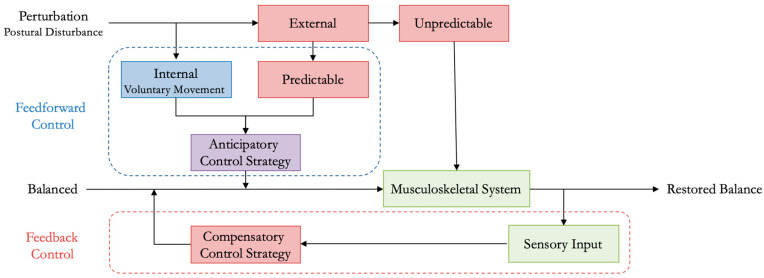
Conceptual model of postural control illustrating the compensatory and anticipatory strategies implemented following unpredictable or predictable perturbations, respectively. Internal perturbations are caused by voluntary movement where specific limbs are moved, whereas external perturbations result from unexpected changes to the sensory environment. During these movements, neighboring skeletal segments may become displaced, affecting overall balance [46,47]. The anticipatory movement strategy implements a feedforward control mechanism, in advance, that predicts the degree of compensation, i.e., adaptation, needed to maintain stability before voluntary movements.

The postural control system consists of complex motor skills coordinated by the CNS that integrate the interaction of multiple sensorimotor processes [44]. Postural control is a learned complex motor skill organized hierarchically by the CNS that constantly adapts and manages the interaction of multiple sensorimotor processes, e.g., visual, vestibular, and somatosensory, derived from an individual’s expectations, goals, cognitive ability, and prior experiences [45]. The failure of this system results in the loss of balance, i.e., instability, which may lead to a fall. The exact mechanism of how the CNS coordinates and regulates postural control is still relatively unknown, as previously alluded to. Postural equilibrium refers to the coordination of sensorimotor strategies to stabilize the body’s COM during internally and externally triggered perturbations to balance [46] (Figure 1). Important extrinsic lower leg muscles involved in maintaining postural equilibrium are listed in Table 2.

Cognitive, sensory, or motor impairments related to aging, injury, neurological disease, and traumatic brain injury create deficits in postural control. Identifying the musculoskeletal, neurological, and pathophysiological changes related to disease is central to understanding the causes and consequences of balance disorders and, thus, their management [47]. Studying balance in healthy and elderly or impaired individuals has provided a significant understanding of the postural control system and its impairments [22,38,48,49,50].

### 1.2. Inverted Pendulum Model and Electromyography

Maintaining a quiet, upright bipedal stance is a fundamental activity of daily living. The single inverted pendulum (SIP) biomechanical model has been accepted as a suitable model to quantify the angle strategy present during the maintenance of balance, i.e., controlling the continuous oscillation of the COP around the COM (postural sway) during quiet standing [39,40,41,42,43,51]. Although passive ankle stiffness has been identified as an important factor in the control of the quiet stance [41], others have suggested that ankle stiffness was insufficient [52,53]. Despite the validation of the SIP, several studies have suggested that the stabilization of the quiet stance is complex, cannot be explained wholly by the SIP model, and is associated with accelerations and movements at both the knee and hip joints [54,55,56,57,58,59,60,61,62,63].

The SIP model’s validation and precision have been widely accepted by the biomechanics community and used extensively as a framework for measuring postural sway in both the antero-posterior (A/P) and medio-lateral (M/L) directions to study the balance of persons with and without musculoskeletal and neurological impairments. Postural sway refers to the involuntary oscillations of the human body during the quiet stance as measured by movements of the COP around the COM. The location of the COP under each foot, with respect to foot orientation, is a direct reflection of the neural mechanisms of postural control [40]. Previous research on quiet bipedal stances has generally focused on determining and examining a variety of metrics related to the COP, e.g., oscillations in the A/P and M/L directions, that might provide insight into postural control [64]. Winter et al. [41,42], whose work has been corroborated by others [21,49,51,65], demonstrated that the ankle dorsi- and plantarflexors partially controlled the A/P oscillations of the COP while loading/unloading at the hip joint controlled M/L oscillations. Other investigators examined COP metrics, as well as selecting lower leg muscle electromyographic (EMG) activity when individuals stood with feet staggered or in tandem [41,66,67,68,69,70,71,72,73,74]. A summary of the results from studies investigating quiet tandem stances demonstrated that, as opposed to the bipedal quiet stance, tandem standing led to (1) an increase in the COP A/P and M/L excursions, which tended to be greater in eyes closed (EC) compared to eyes open (EO) conditions; (2) greater intra- and intertrial (between individuals) variability in COP excursion, yet less so with EO; (3) a greater vertical ground reaction force associated with the rear foot; (4) greater EMG activity in the tibialis anterior (TA), peroneus longus (PL), and soleus (Sol), in general, but greater yet in the rear limb for TA and Sol; (5) greater EMG activity with EC and with an increase in M/L excursion, except for Sol, which was tonically active regardless of changes in COP oscillations; (6) greater cross-correlation of TA and PL, than Sol, with M/L oscillations; and (7) the EMG of the homonymous muscles of the two legs being out-of-phase, which suggests a mutual push-pull action of the muscle pairs. These data suggest that (i) despite large inter-trial and inter-participant variability, the neural commands to the leg muscles during the tandem stance imply a task-sharing rule, whereby the soleus muscles are primarily tasked to keep the body upright while the back-and-forth activity of the TA and PL controls the COM in the frontal plane; (ii) the asymmetry of the mechanical constraints on the feet as a function of the stance organize coordination patterns of feet COPs while the degree of adaptive variation between the feet COPs is dependent on both mechanical and vision constraints; and (iii) asymmetrical loading under each foot may result in different joint compliance properties, i.e., inherent stiffness, that in turn influence the fluctuations of the individual feet COPs and the net COP. This information is valuable because it provides insight into the normal functioning and control of normal quiet balance and provides valid and reliable methods that can be used to assess individuals with musculoskeletal and neurological impairments.

### 1.3. Coherence and Quiet Standing Balance

The shape and firing rate of MUAPs in the EMG signal can potentially provide valuable information about how the CNS coordinates muscle activation, i.e., muscle synergies. As demonstrated in the tandem stance research, increased MUAP recruitment is directly related to increased EMG magnitude. Therefore, a distinct relationship between muscle activation and CNS control must exist that would allow for the indirect extraction of synaptic input signals received by motor neurons from EMG signals. Connectivity analysis methods, such as CMC and IMC, take advantage of the indirect measurement of neural drive within the EMG signals [30,75,76]. The existence of synchronous neural drives can be deduced from comparing IMC between various EMG signals of specific muscles within a functional unit, i.e., synergists. Therefore, the EMG signals of postural control muscles that control quiet bipedal stances may provide valuable insight into how the CNS synchronizes and regulates motor control [77].

Several studies have used IMC analysis to identify synchronization and common neural drive to various postural muscles during a variety of quiet standing tasks in young healthy adults [22,32,33,34,48,49,65,78,79,80,81,82,83,84,85,86,87]. Most studies [22,33,34,48,49,65,81,82,83,84,85] examined common tasks such as quiet bipedal standing; one implemented unipedal [81] or bipedal standing with eyes closed [49,65,80,82] and compared these positions with natural bipedal standing. Other standing tasks included shifting the center of pressure within the base of support [78], dual tasks and standing at a height [22], and tandem stances [83,86,87]. In general, EMG-EMG coherence increased as the base of support narrowed, e.g., unipedal and tandem standing, and the difficulty in standing increased [83,84,86,87]. Significant coherence was observed primarily in the delta band, regardless of the muscle pair [65,79,86], a finding which is thought to reflect synchronous activity of the motor neuron pool [88]. Notably, beta band coherence, which is thought to reflect activity in the corticospinal tract [26,28], i.e., control from the cortex, varied with the complexity of the standing tasks. Since significant beta band coherence was demonstrated in standing tasks characterized by a narrow base of support [83,86,87], the cortical control of standing may increase as the difficulty of the standing task increases. Two studies reported increased coherence during eyes closed conditions [48,80,86], while others reported no change [65] or the opposite effect [48]. Two studies [48,81] investigated muscle pairs across joints according to M-mode components. The delta band was the most common frequency band of interest for coherence analysis, although many studies also examined alpha, beta, and gamma bands. Yet, the specific frequency band values were not consistent. Most studies selected 0–5 Hz for the coherence analysis of the delta band, and approximately 50% of the studies chose 15–35 Hz for the beta band [77].

Many other studies compared intermuscular coherence analyses of quiet standing tasks between young and elderly healthy adults [50,80,89,90,91,92,93,94], where significant differences were noted between the two age groups. For example, Obata et al. [80] reported a significant coherence in 0–4 Hz for both groups in bilateral and unilateral plantarflexors, while a coherence in 8–12 Hz was found only for the elderly group in bilateral muscle pairs. Watanabe et al. [92] also found that during bipedal standing, the delta band coherence of the plantarflexor pairs was greater in elderly adults and that the delta band coherence was greater in unipedal than in bipedal stances for both age groups, but greater in elders compared to younger adults. Degani et al. [90] determined the IMC of synergistic muscle groups and found that young adults presented with significant coherence in the 0–5 Hz frequency band in the soleus and biceps femoris pair, whereas the older adults presented with coherence in the 0–10 Hz frequency range. These studies have also clearly shown that EMG-EMG coherence was related to increased postural sway during standing in elders and that older adults appeared to selectively increase the corticospinal drive to lower leg muscles to cope with increased postural sway.

In the past two decades, the use of IMC has been used extensively in attempts to clarify neural mechanisms related to the postural control of the quiet stance in young and elderly healthy adults. Most of these studies focused their analysis on normal bipedal standing postures, but only two examined the EMG-EMG coherence of paired lower leg muscles during tandem standing. The primary aim of this study was to extend the work of Nandi et al. and Ohja et al. [83,86] and examine how the functional connectivity of lower leg muscle pairs would change during increased levels of standing postural instability. Although [86] demonstrated that muscle pairs in the rear leg demonstrated greater connectivity, the role of the dominant leg was not examined. Therefore, secondarily, we assessed the role of the dominant leg in maintaining tandem standing balance.

## 2. Materials and Methods

### 2.1. Participants

Eight healthy young adults (age: 24.8 ± 3.3 years; height 171.0 ± 10.5 cm; body mass: 71.0 ± 13.5 kg) volunteered following informed consent; however, only six individuals (2 males and 4 females), aged 18–34, and of varying physical activity levels, were included in the final data analysis. All participants were considered healthy without a history of neurological or muscular disorders or recent previous injuries. Before data collection commenced, leg/foot dominance for each participant was determined based on the leg with which they preferred to kick a ball. A follow-up task of standing on one leg was implemented for participants who were unable to determine a preference from the previously asked questions. Three participants were right-leg and three left-leg dominant. The Human Research Review Committee, Institutional Review Board, Office of Research Compliance and Integrity, at Grand Valley State University approved this study (18-246-H).

### 2.2. Experimental Protocol

Participants completed five 30 s trials of six different balancing conditions (Table 3) starting with eyes open, feet together (EOFT) to quantify a stable baseline to compare to other balance conditions. A 30 s break was implemented between trials, alongside a 2 min break between each condition. Test conditions were not randomly ordered but were completed in the order they were listed across all participants. Balance tasks were performed barefoot, and arms were positioned with the shoulders flexed slightly and elbows fully flexed so that the index finger pointed towards the ipsilateral shoulders.

### 2.3. Data Acquisition

Surface electromyographic (EMG) signals (1200 Hz), motion trajectories (120 Hz), and ground reaction forces (1200 Hz; Advanced Mechanical Technology Inc., Watertown, MA, USA) were synchronized using Vicon NEXUS motion capture software v2.8 (Oxford Metrics, Oxford, UK). Only EMG data were used for analysis in this study.

Electrical activity was recorded from the left (L) and right (R) tibialis anterior (TA), medial gastrocnemius (MG), and soleus (S) muscles [95]. These muscles were chosen because of their prominent role in standing postural control [39,40,43]. Before the application of the surface electrodes, the skin of the lower legs was shaved to remove excessive hair and was cleaned with rubbing alcohol. Electrodes were secured to the skin with hypoallergenic tape and circumferentially secured using sports prewrap tape. Electrodes (interelectrode distance of 17 mm) were placed parallel to the muscle fiber direction by the primary researcher according to SENIAM recommendations [96] and were supervised by the laboratory research director, who had 20 years of experience. Accurate electrode placement was verified by performing manual muscle tests for each muscle. A reference, i.e., ground, electrode was secured to the patella on one of the lower extremities.

The MA-411 pre-amplifiers (double differential with Common Mode Rejection Ratio > 100 dB at 65 Hz, noise < 1.2 µV, and input impedance > 100,000 MΩ) were used to record data, which incorporate both radio frequency interference filters and electrostatic discharge protection circuitry that helps to eliminate motion artifacts and cable noise, providing the most reliable EMG signal (Motion Lab Systems Inc., Baton Rouge, LA, USA). The pre-amplifiers were interfaced with the MA300-XVI EMG patient unit acquisition system. The patient unit of the MA-411 implemented a 500 Hz low-pass anti-aliasing filter on the raw EMG before transmitting it to the desktop unit where the signal was further filtered with a 10 Hz high-pass filter. Raw EMG signals from each muscle for one representative participant were similar to those from a previous study that used similar methods (see Figure 1 in Ojha et al. [86]). All recorded EMG signals were analyzed in the frequency domain using MATLAB R2018a (The MathWorks, Natick, MA, USA) for the following neural frequency bands (Table 4) and muscle pairs (Table 5) to observe the presence of synchronized correlated neural drives.

### 2.4. Data Analysis

MATLAB’s Welch’s power spectral density (PSD) estimator was used to visually analyze the frequency content of the raw 30 s EMG data collected for the baseline condition, for all muscles, and for each subject to identify any noise artifacts. A 60 Hz 2nd-order Butterworth notch filter with a 0.2 Hz bandwidth was used to remove the powerline interference at 60 Hz.

Magnitude-squared coherence (MSC) measures the linearity of the phase relation between two signals, *x* and *y,* in the frequency domain, defined by

$$C_{xy}=\frac{{\left|P_{xy}\left(f\right)\right|}^{2}}{P_{xx}\left(f\right)P_{yy}\left(f\right)}$$

$C_{xy}$ is MSC, $P_{xy}$ is the cross-spectrum power, and $C_{xx}$ and $C_{yy}$ are the auto-spectrums of input signals *x* and *y* at frequency *f*. MSC values range between 0 and 1, where 0 indicates no linear relationship and 1 is a perfect linear relationship. MSC was estimated between pairs of EMG signals and was used to quantify IMC, providing insight into the connectivity between EMG signals of neighboring leg muscles.

**Figure 2:**
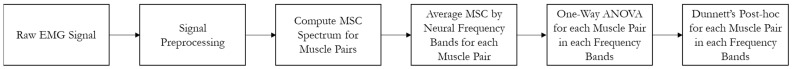
Steps for data processing for each participant.

MSC was calculated from MATLAB’s built-in MSC function that estimates $C_{xy}$ using Welch’s overlapped periodogram method. The MSC spectrum was estimated for the whole 30 s of all filtered EMG data for each muscle pair listed in Table 3 across the neural frequency range (0–100 Hz) for each standing condition and each participant. Magnitude-squared coherence was estimated from a two-second Hamming window, with a 25% overlap. This created 19 window segments of 2400 data points and a frequency resolution of 0.5 Hz. Each neural frequency range was averaged across its MSC spectrum to generate a singular coherence value for that range. The functional block diagram for data and statistical analysis is illustrated in Figure 2.

### 2.5. Statistical Analysis

Statistical analysis and graphics were performed with R Statistical Software (v4.4.0; R Core Team 2024,Vienna, Austria) [97] running in RStudio: Integrated Development Environment for R [98]. Raw data for each set of five independent trials across the six test conditions within the nine muscle pairs and six frequency bands for each participant were examined for the magnitude and variation in coherence measurements. We noted that the 10 Hz high-pass filter masked significant EMG-EMG coherence in the delta and theta frequency bands, so these data were dropped from the main analysis. Based on observations of the full data set, we also determined that data from two muscle pair groups would not be included in our final analyses due to insufficient EMG connectivity. The resulting charts of five independent trials across the six test conditions within the seven muscle pairs and four frequency bands for each of the six subjects are included in the Appendix A, with only participant #3 (PO3) data being included in Section 3.1 (Figure 10). A single graphic (Figure 10) with all data from this reduced set of data were generated to help visualize inter-trial and inter-participant variation. Analysis was performed on 168 sets of 30 coherence values from the six conditions and five independent trials within each participant, frequency band, and muscle group. The primary goal of the analysis was to determine under what conditions the mean coherence differed from the baseline EOFT condition. This implied the use of a Dunnett’s test. Assumptions of normality (Shapiro tests) and homogeneity of variance (Levene’s test) were performed on each data set with failures of assumptions graphed using Q-Q plots and boxplots. Given the exploratory nature of this study and the mild nature of deviations from the statistical assumptions, we chose to apply parametric methods without transformation for simplicity. Dunnett’s test results are summarized graphically with an array of 95% Dunnett’s confidence interval charts (Figures 4–9), each showing the interval for the mean difference between the less stable condition (ECFT, EOTanDB, ECTanDB, EOTanDF, and ECTanDF) and the baseline EOFT condition, i.e., the interval for the increase in coherence for each of the less stable conditions. Intervals associated with a significant result (*p*-value < 0.05) are colored blue. Note: ANOVA F-tests were performed but rejected too frequently, i.e., a significant F-test often did not result in any significant Dunnett’s interval. This is because the F-test looks at all possible pairs of conditions, not just the 5 comparisons of interest but all 15 possible combinations.

## 3. Results

### 3.1. Overview of EMG-EMG Coherence Across Participants

After reviewing the magnitude-squared coherence for each muscle pair and all participants across the test conditions and frequency bands, we focused the analysis on the alpha, beta, lower gamma, and upper gamma frequency bands. We eliminated the analysis of the delta and theta frequency bands because we believed that the default low- and high-pass filters built into the EMG pre-amplifiers resulted in the loss of meaningful data. Moreover, the left medial gastrocnemius–right medial gastrocnemius and left soleus–right soleus muscle pair MSC data were not part of our final analysis. This section will present an overview of the muscular coherence patterns across participants, based on our observations of the results. Because of the variability of the muscular coherence data (details to be discussed in Section 3.3), we cannot illustrate overall patterns from a representative participant. However, we will provide the muscular coherence data from participant #3 to assist the reader in a review of all participant data, which can be found in the Appendix A.

**Figure 3:**
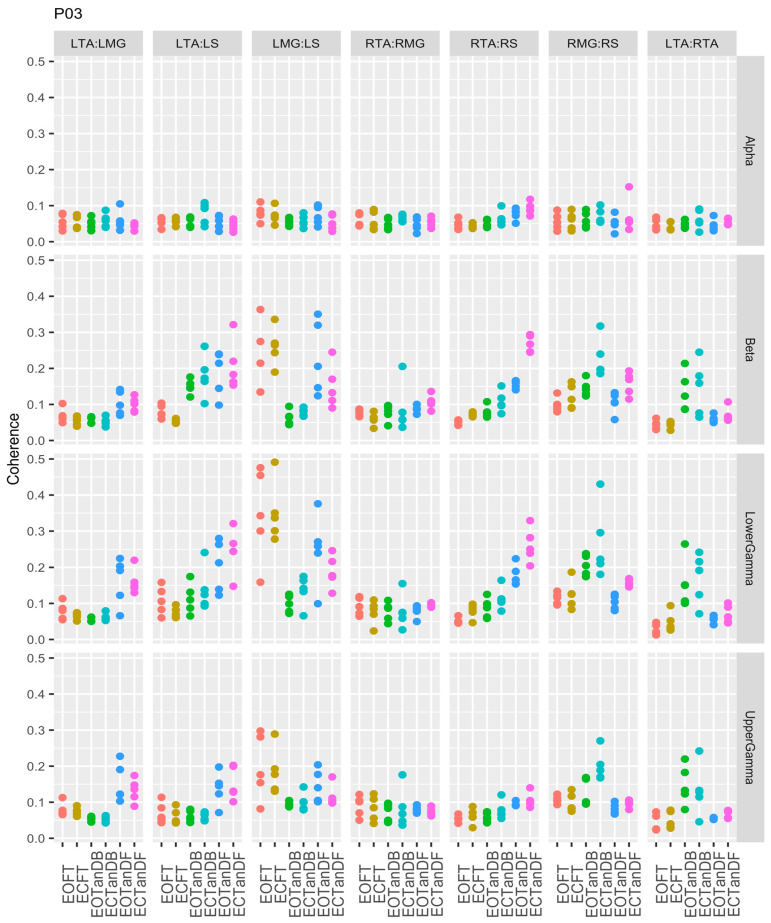
EMG-EMG muscular coherence for participant #3 (P03) for five trials over six conditions (EOFT = eyes open feet together; ECFT = eyes closed feet together; EOTanDB = eyes open tandem dominant leg back; ECTanDB = eyes closed tandem dominant leg back; EOTanDF = eyes open tandem dominant leg forward; ECTanDF = eyes closed tandem dominant leg forward) for all muscle pair combinations across four frequency bands: alpha (8–13 Hz), beta (13–30 Hz), lower gamma (30–60 Hz), and upper gamma (60–100 Hz). Note: LMG/RMG = left and right medial gastrocnemius; LS/RS = left and right soleus; LTA/RTA = left and right tibialis anterior.

The qualitative-based analysis noted the following EMG-EMG muscular coherence patterns (Figure 3):

- Consistently greater coherence between muscle pairs in the tandem stance postures compared to the feet together stance postures;
- Consistently greater coherence in the LMG: LS and RMG: RS muscle pairs across the beta, lower gamma, and upper gamma frequency bands for tandem stance postures in both eyes open and closed conditions, without discernable differences between eyes open and eyes closed;
- Demonstrable evidence of coherence between antagonistic muscle pairs, e.g., LTA: LS, primarily in the tandem stance postures.

### 3.2. Comparison of EMG-EMG Muscular Coherence Across Conditions

The primary purpose of this project was to examine whether muscular coherence differed between a baseline standing posture (EOFT) and less stable standing postures, e.g., tandem standing postures with eyes open and closed. A Dunnett’s inferential analysis demonstrated selected significant differences in muscular coherence between more and less stable standing postures across select muscle pairs and test conditions. In this section, we will present data from all six participants and summarize our major findings.

**Figure 4:**
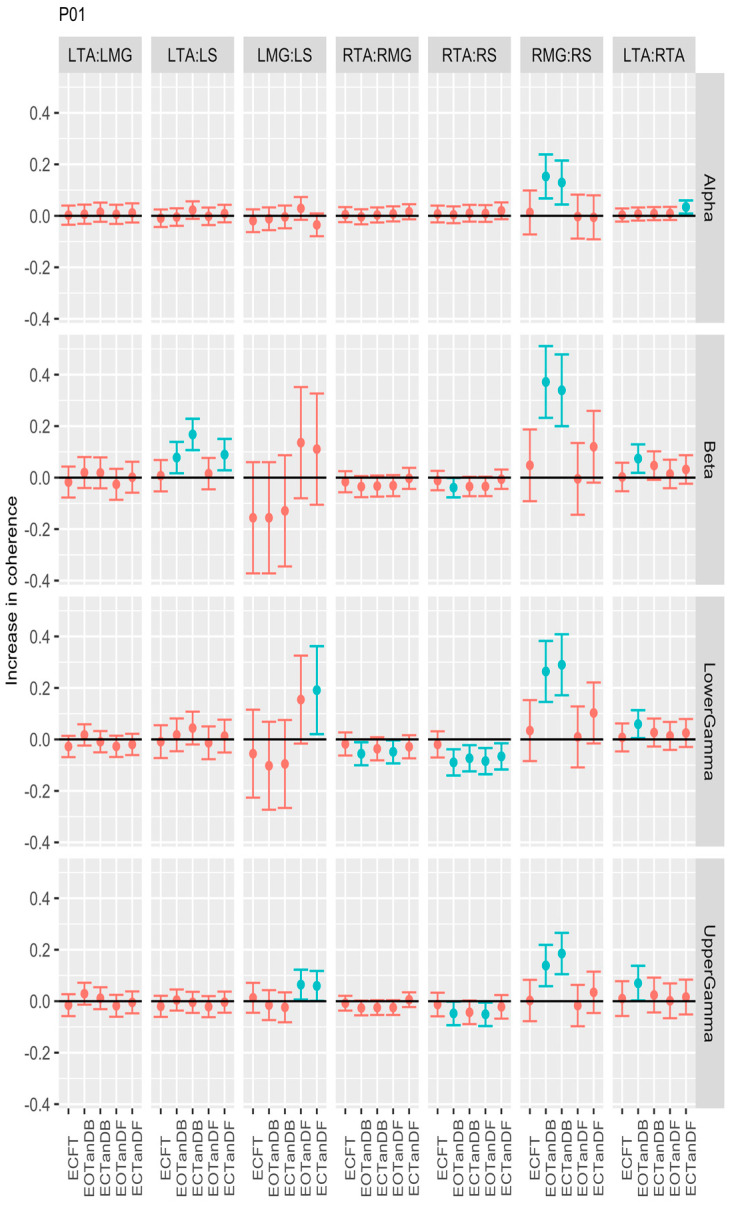
Confidence intervals (CIs) for mean differences in coherence between less stable, e.g., EOTanDB, standing postures and the baseline standing posture, i.e., EOFT for participant #1 (PO1). Differences and their confidence intervals (CIs) are presented as red (no significant difference) and blue (significantly different). The black horizontal line indicates zero difference, CIs above zero indicate that the test condition was greater than the baseline, and CIs below zero indicate that the test condition was less than the baseline.

For participant #1, there was a consistent difference between tandem standing (dominant leg back) and the baseline condition, with greater coherence for the RMG: RS muscle pair across all frequency bands. Surprisingly, greater coherence for the LMG: LS muscle pair was only evident for a couple of test conditions in the lower and upper gamma frequency bands (Figure 4).

**Figure 5:**
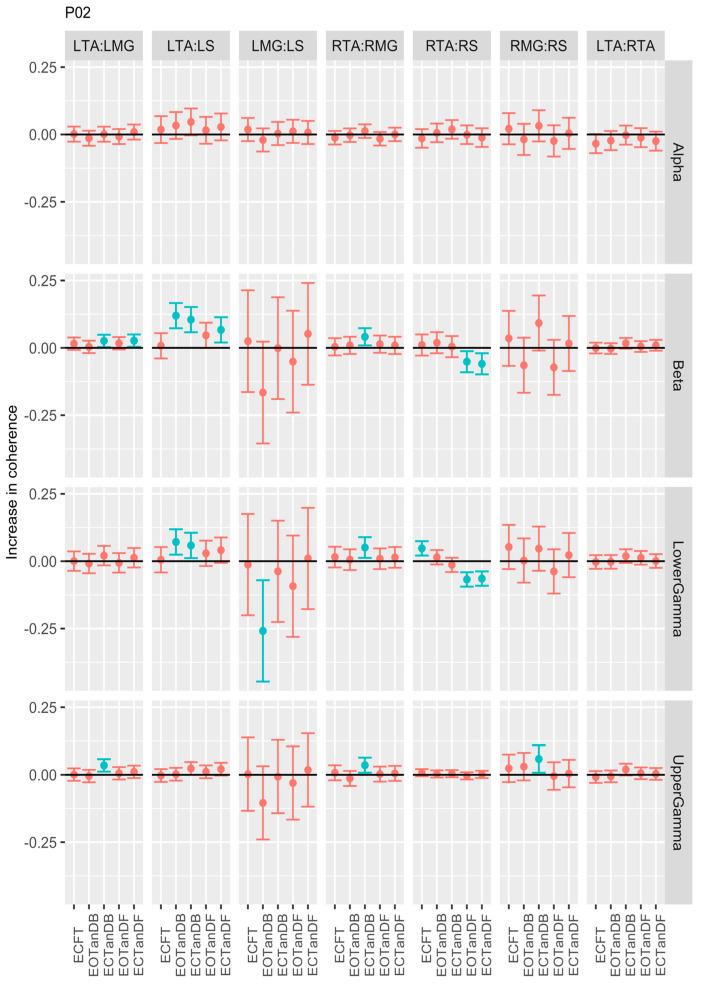
Confidence intervals (CIs) for mean differences in coherence between less stable, e.g., EOTanDB, standing postures and the baseline standing posture, i.e., EOFT for participant #2 (PO2). Differences and their confidence intervals (CIs) are presented as red (no significant difference) and blue (significantly different). The black horizontal line indicates zero difference, CIs above zero indicate that the test condition was greater than the baseline, and CIs below zero indicate that the test condition was less than the baseline.

Participant #2 demonstrated different patterns. For instance, (1) there were no differences in coherence in the alpha frequency band, (2) greater coherence in less stable standing postures for LTA: LS muscle pair was seen in the beta band, and (3) a mix of greater and lesser coherence in less stable standing postures for the RTA: RMG and RTA: RS in the beta and lower and upper gamma bands was observed. However, no differences in coherence in the LMG: LS and RMG: RS muscle pairs were found in the less stable postures, as we saw for P01 (Figure 5).

**Figure 6:**
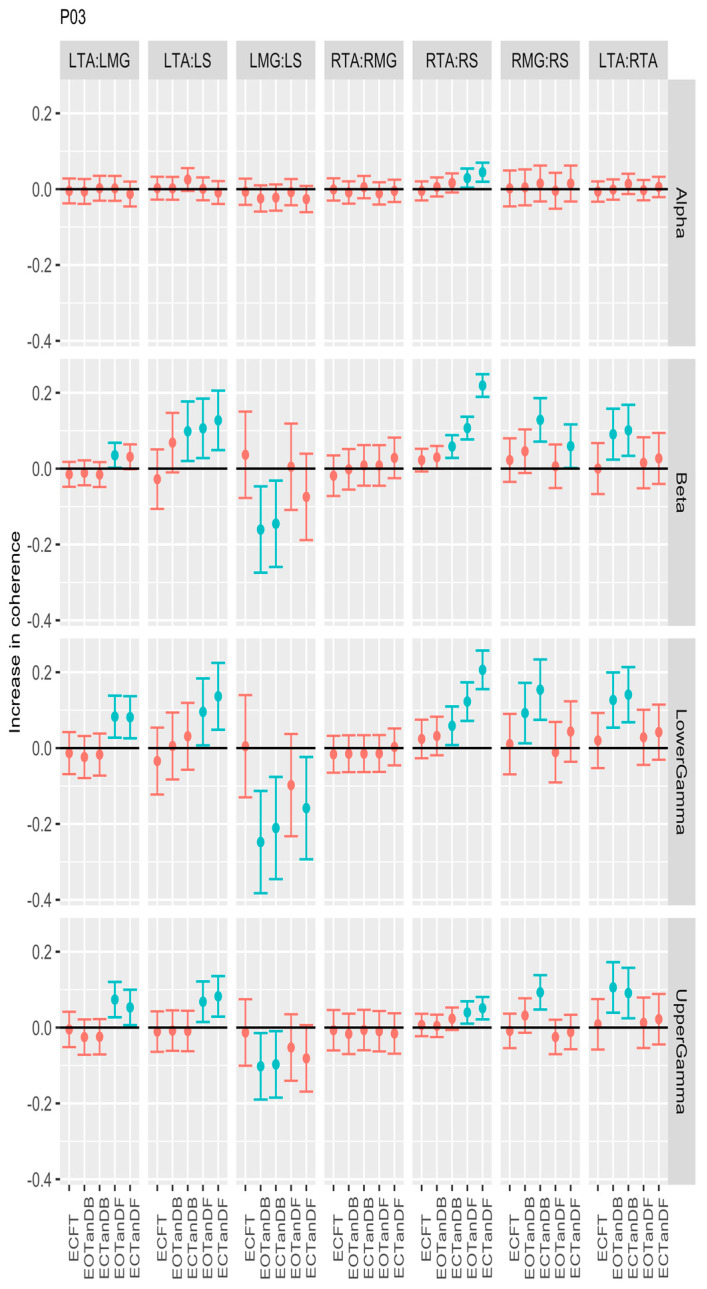
Confidence intervals (CIs) for mean differences in coherence between less stable, e.g., EOTanDB, standing postures and the baseline standing posture, i.e., EOFT for participant #3 (PO3). Differences and their confidence intervals (CIs) are presented as red (no significant difference) and blue (significantly different). The black horizontal line indicates zero difference, CIs above zero indicate that the test condition was greater than the baseline, and CIs below zero indicate that the test condition was less than the baseline.

**Figure 7:**
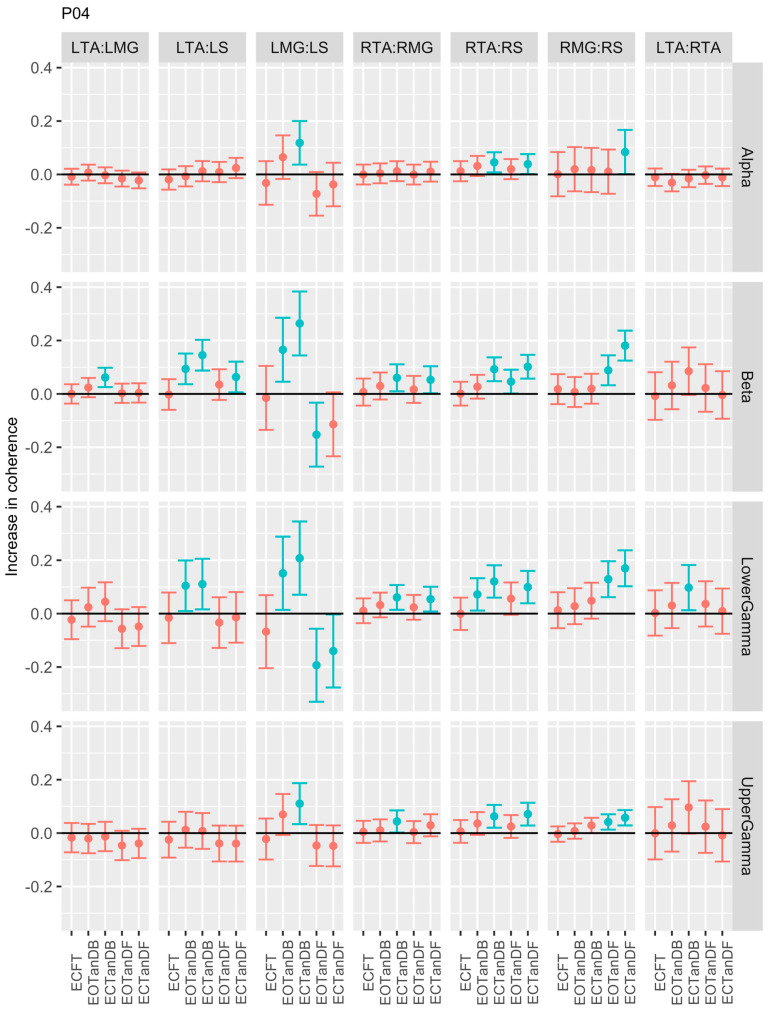
Confidence intervals (CIs) for mean differences in coherence between less stable, e.g., EOTanDB, standing postures and the baseline standing posture, i.e., EOFT for participant #4 (PO4). Differences and their confidence intervals (CIs) are presented as red (no significant difference) and blue (significantly different). The black horizontal line indicates zero difference, CIs above zero indicate that the test condition was greater than the baseline, and CIs below zero indicate that the test condition was less than the baseline.

For participants #3 (Figure 6) and #4 (Figure 7), we observed significant differences in coherence between the less stable standing postures and the baseline for six muscle pairs, primarily in the beta, lower gamma, and upper gamma frequency bands. Unlike P01, which demonstrated greater coherence in the LMG: LS and RMG: RS muscle pairs, P03 showed less coherence in the LMG: LS muscle pair but greater coherence in the RMG: RS muscle pair. However, P04 showed inconsistent coherence differences in the LMG: LS muscle pair when compared to P03. P03 and P04 consistently showed greater coherence in the LTA: LMG, LTA: LS, RTA: RS, and LTA: RTA muscle pairs in the beta and lower and upper gamma frequency bands.

**Figure 8:**
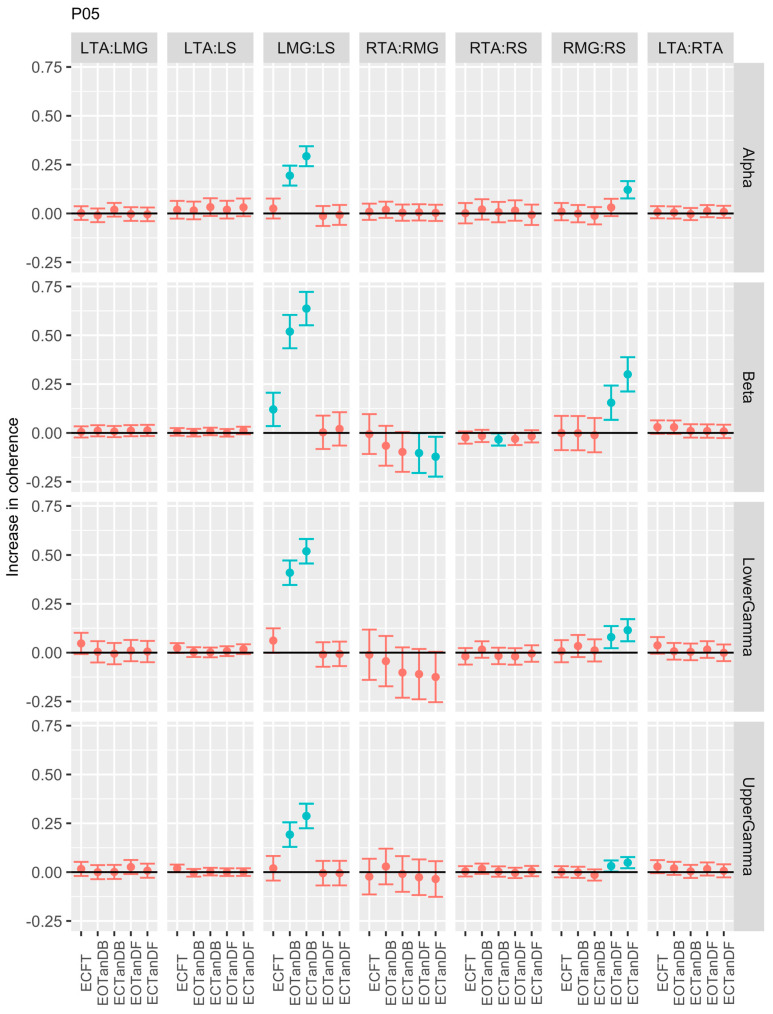
Confidence intervals (CIs) for mean differences in coherence between less stable, e.g., EOTanDB, standing postures and the baseline standing posture, i.e., EOFT for participant #5 (PO5). Differences and their confidence intervals (CIs) are presented as red (no significant difference) and blue (significantly different). The black horizontal line indicates zero difference, CIs above zero indicate that the test condition was greater than the baseline, and CIs below zero indicate that the test condition was less than the baseline.

**Figure 9:**
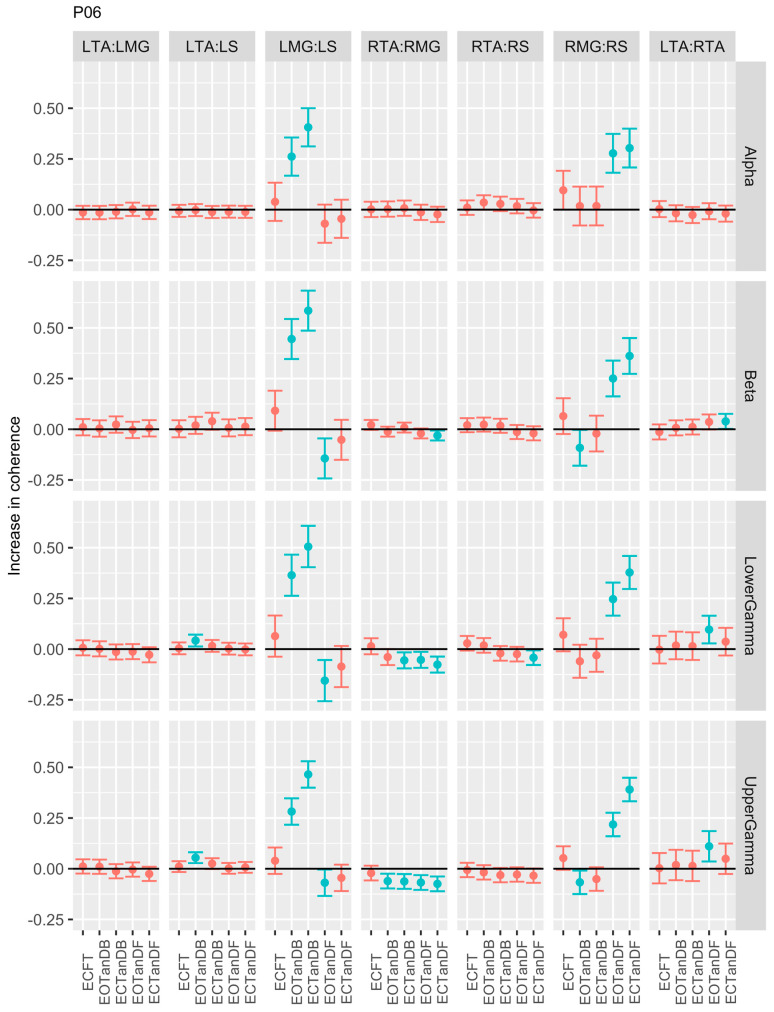
Confidence intervals (CIs) for mean differences in coherence between less stable, e.g., EOTanDB, standing postures, and the baseline standing posture, i.e., EOFT for participant #6 (PO6). Differences and their confidence intervals (CIs) are presented as red (no significant difference) and blue (significantly different). The black horizontal line indicates zero difference, CIs above zero indicate that the test condition was greater than the baseline, and CIs below zero indicate that the test condition was less than the baseline.

Participants #5 (Figure 8) and #6 (Figure 9) were similar to P01 in demonstrating greater coherence primarily in the LMG: LS and RMG: RS muscle pairs for less stable standing postures across all frequency bands. P05 showed reduced coherence only for the RTA: RMG muscle pair in tandem standing in the beta band, whereas P06 showed reduced coherence for the same muscle pair in the beta and lower and upper gamma bands. Participant #6 was distinguished from P05 in that there was a trend toward greater coherence in the LTA: RTA muscle pair in less stable standing postures in the beta and lower and upper gamma bands.

In summary, from Section 3.1 and Section 3.2, (1) generally, there appeared to be notable differences between the six participants in terms of how the coherence of muscle pairs changed in the tandem, i.e., less stable, standing postures; (2) muscular coherence significantly increased in selected muscle pairs in the tandem standing postures; (3) the most common muscle pairs affected by the tandem standing postures were LMG: LS and RMG: RS; (4) muscular coherence between antagonistic muscles, e.g., LTA: LS, during the tandem standing tasks suggests a more complex motor control pattern during less stable standing postures; (5) there did not appear to be differences in changed muscular coherence in the tandem eyes open and eyes closed conditions; and (6) changed muscular coherence in muscles pairs did not appear to be different when the dominant leg was placed on the rear or front forceplate.

### 3.3. Intertrial and Intersubject EMG-EMG Muscular Coherence Variability

**Figure 10:**
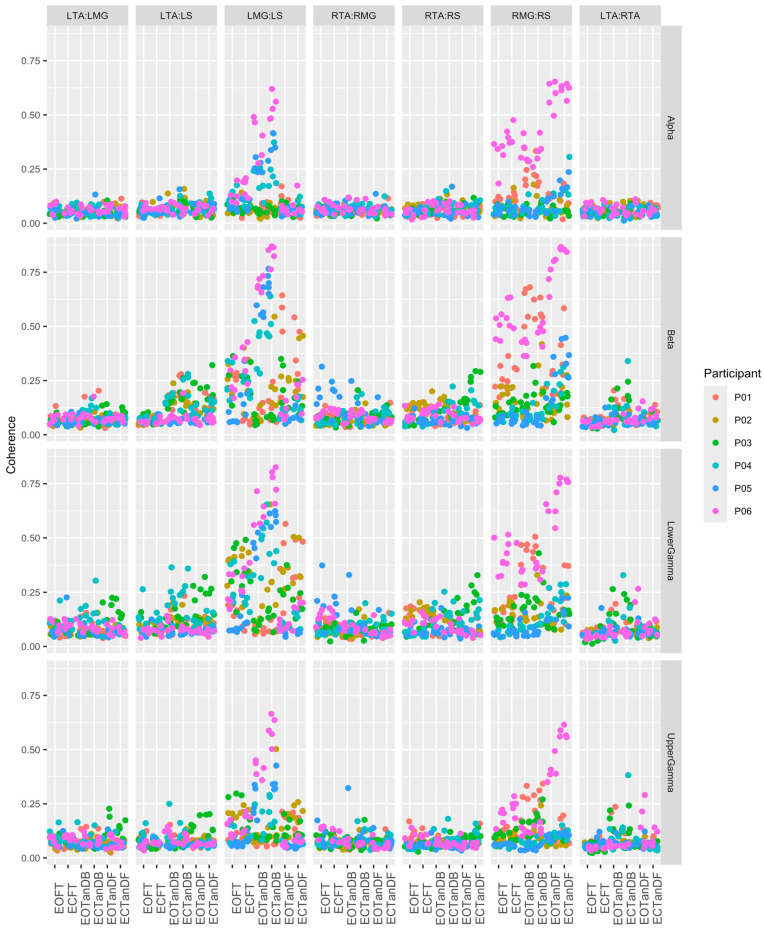
Magnitude-squared coherence for all six participants across five trials, and all muscle pairs for the alpha, beta, and lower and upper gamma frequency bands.

Previously, we identified inter-trial and inter-participant variability (Appendix A), and in Section 3.2, we noted differences in muscular coherence patterns across the six participants. In Figure 10, we illustrated the notable biological variability as individuals adapted to changes in standing postures. The greatest variability is seen in the LMG: LS and RMG: RS muscle pairs across all frequency bands. As it turns out, these were the muscle pairs most affected by the less stable standing postures. Figure 10 also shows that P06 appeared to have the most variability between trials, particularly for the LMG: LS and RMG: RS muscle pairs.

## 4. Discussion

A significant incidence of falls related to the effects of aging and degenerative and neurological diseases is a major problem worldwide [3,4,5,6,7,8,9,10,11]. Since falls have been associated with a reduced ability to control postural sway [1,2], acquiring insight into the neurological mechanisms of standing balance and postural control is important. Previous research has addressed many aspects of biomechanical and central nervous systems’ control of postural sway for both normal and elderly individuals based on the inverted pendulum model [39,40] for natural quiet standing postures [19,22,33,45,46,65,80,89], as well as the more difficult tandem stance [71,83,86,87]. In tandem standing, it has been shown that the EMG-EMG coherence of selected lower leg muscles is greater than natural standing and that larger vertical ground reaction forces exist under the rear foot. However, it is unclear what role the dominant leg/foot plays in maintaining a tandem standing posture. Having additional insight on tandem standing in healthy individuals is important to help our baseline understanding of different standing postures, because this posture can be used as a tool to test those with musculoskeletal and neurological diseases who have impaired balance. Therefore, this project examined (1) how the functional connectivity of lower leg muscle pairs, i.e., EMG-EMG coherence, would change with greater standing posture challenges and (2) the role of the dominant leg in maintaining tandem standing posture. Our results showed consistently greater coherence in the beta, lower gamma, and upper gamma frequency bands, particularly between the medial gastrocnemius and soleus muscle pairs bilaterally in the tandem stance posture, without discernable differences between eyes open or closed conditions. Although the EMG-EMG coherence of muscle pairs was typically greater in the rear leg, functional connectivity did not appear to be different when the dominant leg was in the rear or fore position during tandem standing.

In his seminal work on the coordination and regulation of movement, Berstein [13] highlighted the complexity of the CNS and its challenge to manage the degree of freedom (DoF) redundancy. Bernstein’s hierarchical control theory suggested that the CNS governed specific functional control structures, which may minimize the DoF problem [13,15,99]. Latash and colleagues [16,17,18,100] acknowledged the DoF issue and, noting the variability in human movement, claimed that a DoF problem did not exist. Their alternative, called the “principal of abundance”, suggested that all the elements, i.e., DoFs, always participated in all tasks, so that performance stability and flexibility, i.e., variability, could be optimized depending on the functional demands. Latash et al.’s [16,100] uncontrolled manifold hypothesis (UCM) was an attempt to define muscle synergies better and provide a method to examine the control of movement patterns related to an infinite number of activities. Krishnamoorthy et al. [19,20] examined shifts in the center of pressure and the EMG activity of several postural muscles, using the UCM to explore the role of muscle synergies during different standing tasks. They identified three muscle synergy patterns, i.e., muscle (or M) modes, that involved the control of antero-posterior postural sway. Other methods using the EMG of postural muscles and principal component analysis (PCA) and/or non-negative matrix factorization (NNMF) [21,22] have also attempted to refine the ability to identify muscle synergies related to the control of quiet standing posture. In the last three decades, this work segued to research that used the EMG signal to examine the spectral information about motor neuron firing and the motor unit action potentials [101,102]. Thus, common presynaptic inputs to motor neuron pools of two or more muscles can synchronize their firing frequency, where the strength of such synchronization becomes apparent in the coherence, i.e., a measure of correlation in the frequency domain, between trains of action potentials discharged by motor neurons innervating two muscles. Common neural inputs to different muscles can then be inferred based on intermuscular (IMC or EMG-EMG) methods to examine the role of common neural drives that synchronously activate motor pools of individual muscles in a functional synergy [25,26,27,28,29,30,31].

If, from a neurological perspective, standing balance is maintained by functional synergies through the coordinated action of lower leg muscles, we assume that neural control may be simplified by the synchronized, i.e., common inputs, activation of muscles comprising a functional synergy as a single unit, rather than separate neural signals to each muscle [25,49,79,101]. From a biomechanical perspective, the single inverted pendulum (SIP) model, using the EMG of lower leg muscles, has been used to study quiet stance [39,40,41,42,43,51]. These studies showed that during a natural quiet stance, i.e., standing with feet apart, the antero-posterior postural sway is controlled primarily by the lower leg muscles, e.g., dorsi-and plantarflexors, whereas medio-lateral sway is controlled by the ankle invertors/evertors and loading/unloading at the hip joint. While the results of this early work provided valuable insights into an aspect of postural control, it insufficiently did so because of methodological limitations. Research using IMC based on the SIP has significantly increased our understanding of quiet standing postural control, under a variety of conditions, in young [22,32,33,34,48,49,65,78,79,80,81,82,83,84,85,86,87] and older adults [50,80,89,90,91,92,93,94] by providing information on the neural inputs to muscles at different frequencies that are characteristic of activity in different areas of the central nervous system, i.e., cortical, subcortical, or spinal. Therefore, the source of presynaptic common inputs can be inferred based on the frequencies at which EMG-EMG coherences have emerged [26]. For example, during quiet standing, the coherence of select lower leg muscle pairs has been reported in the 0-5 Hz (delta), 6-15 Hz (alpha), and 13-30 Hz (beta) bands. Delta and alpha frequency bands are thought to reflect subcortical inputs [80,101], and although alpha frequency bands may also involve corticospinal contributions, the beta band is primarily thought to reflect corticospinal control [77]. As the standing task difficulty increases, e.g., tandem stance, significant beta band coherence is evident [83,86,87], and the corticospinal excitability of the lower leg muscles has also been shown to increase [103].

Since our research involved two standing tasks more difficult than natural quiet standing, i.e., feet together and tandem, under both eyes open and closed conditions, it is most relevant to discuss our findings relative to previous research that examined the EMG-EMG coherence of lower leg muscles during quiet tandem standing postures [83,86,87]. Although we collected ground reaction force data in our cohort, we have not presented those results here. However, in a previous publication using data from the same cohort as ours, Tipton et al. [104] showed that the approximate entropy (ApEn) and velocity of the center of pressure (COP) in both the antero-posterior and medio-lateral directions were significantly increased in three less stable (more difficult) standing positions: feet together eyes closed and tandem with both eyes open and eyes closed. An increase in ApEn suggests a more variable time series and less predictable pattern associated with a novel movement pattern, e.g., tandem standing. The COP findings in Tipton et al.’s report are consistent with many previous studies on the biomechanical and neurological challenges of tandem standing in non-impaired, healthy adults [41,66,67,68,69,70,71,72,73,74].

Ojha et al. [86] studied healthy young adults using a similar protocol to ours. That is, they synchronized the motion capture of various standing barefoot postural stances and the collection of the ground reaction forces and EMG of the tibialis anterior (AT), soleus (S), and medial gastrocnemius (MG) muscles bilaterally. Their tandem stance data, however, only reflected when the dominant foot was on the rear forceplate. Ojha used wavelet decomposition to extract the neural frequency bands, namely delta, theta, alpha, beta, and gamma, and used magnitude-squared coherence to examine common neural inputs to co-acting muscle pairs bilaterally: LAT-LMG, LAT-LS, LMG-LS, RAT-RMG, RAT-RS, LAT-RAT, LMG-RMG, AND LS-RS (where L = left and R = right). Tests for EMG cross-talk between muscle pairs demonstrated that cross-talk was negligible. They showed that with increased postural challenges, i.e., eyes closed and tandem stances, the EMG signal density increased in all muscles, and that coherence between muscle pairs trended higher in the least stable stances. There was greater coherence between muscle pairs of the same leg in the lower frequency band, i.e., delta, particularly for the right, i.e., dominant, limb. Furthermore, coherence between RMG and RS was greater under all conditions [86].

Our study extended Ojha et al.’s results and tested a potential role the dominant limb might play in maintaining postural stability in the same postural conditions. Although we did not analyze the coherence in the delta and theta frequency bands, our results showed increased coherence between all muscle pairs in the tandem stance position compared to the baseline (or control) posture, i.e., feet together eyes open. Moreover, there was consistently greater coherence in the LMG: LS and RMG: RS muscle pairs across the beta, lower gamma, and upper gamma frequency bands for tandem stance postures in both the eyes open and eyes closed conditions, although there were no discernible differences in coherence when the eyes were open or closed. Kerkman et al. [32] also reported increased connectivity at higher frequency components (11–21 Hz and 21–60 Hz), but within and between lower leg and torso synergies. They suggested that higher frequency components may reflect propriospinal pathways. Our coherence results in the beta and gamma frequency bands were different than what Ojha et al. reported, suggesting that future research might attempt to replicate our findings with a larger sample. We also reported coherence between antagonistic muscle pairs, e.g., LTA: LS, primarily in the tandem stance postures. This suggests that increased stiffness produced by these co-actions may be needed for less stable postures. When we looked at the possible role of the dominant limb, it was apparent that muscle pair coherences were not different when the dominant limb was placed over the rear or forward forceplate. Finally, perhaps consistent with Latash et al.’s principle of abundance [16,17], we identified consistent inter-trial and inter-participant variability in muscle coherence patterns across our six participants, which was similar to what Torres-Oviedo and Ting reported [21,105]. These results may be indicative of the CNS’s ability to use all the elements, i.e., DoFs, in all of the postural tasks used in this project to ensure both flexible responses and standing stability.

In another comparable study, Nandi et al. [83] collected ground reaction force (COP) and surface EMG from six muscles, on the dominant side only, (soleus, Sol; lateral gastrocnemius, LG; tibialis anterior, TA; peroneus longus, PL; biceps femoris, BF; rectus femoris, RF) under four conditions (standing in stocking feet), in random order: (1) tandem stance with the dominant foot posterior, (2) wide stance (feet shoulder width apart), (3) narrow stance (feet together), and (4) one leg stance (dominant foot). Single-pair EMG-EMG coherence was estimated for the agonist–agonist (AG-AG) pairs: Sol-LG, Sol-PL, and LG-PL, and the agonist–antagonist (AG-ANT) pairs: Sol-TA, LG-TA, PL-TA, and RF-BF in the 0–55 Hz range, thus reflecting subcortical/spinal and corticospinal inputs at 0–5 and 6–15 Hz and 6–15 and 16–40 Hz, respectively. They found that coherence was greater in the AG-AG compared to the AG-ANT muscle pairs in all frequency bands, suggesting that the system preferred functional synergies consistent with reciprocal rather than stiffness control. Coherence increased with stance difficulty but only in the AG-ANT muscle pairs in the delta band (0–5 Hz), reflecting subcortical input, whereas greater coherence in the AG-AG group, with increasing postural challenges, was noted in the beta and lower gamma bands (16–40 Hz), reflecting corticospinal inputs. Although we did not examine the coherence of the AG-ANT invertor/evertor antagonist muscle pair, e.g., PL-TA, our findings and conclusions are similar to Nandi et al. [83].

Tsiouri et al. [87] suggested that although the role of large muscle groups in postural control was substantial, the contribution of the toe flexors was underappreciated. To date, they have been the only group to examine the coherence of both extrinsic, i.e., ankle, and intrinsic foot muscles in the control of standing balance. They included young healthy adults, collecting the ground reaction forces (COP) and EMG activity of the flexor digitorum brevis (FDB), soleus (SOL), medial gastrocnemius (MG), and tibialis anterior (TA) while barefoot under four stance conditions: bipedal, tandem, one-legged (dominant leg), and on toes. The coherence of rectified EMG signals in the 0–60 Hz band, but with two frequency bands of interest, i.e., 0–5 Hz and 10–20 Hz, was determined for the SOL/FDB muscle pair. They showed that COP sway and the EMG activity in all muscles were greater for the three more difficult postural stances and that significant coherence between the SOL and FDB was found in both frequency bands. Notably, greater coherence with increased stance challenges was greater in the 10–20 Hz (or beta) band. Their findings are consistent with previously cited papers and underscore the relationship between the FDB and soleus muscles and the importance of the FDB muscles in the control of standing balance.

It may be that the research that has used traditional analyses and methodologies combining three-dimensional (3D) motion capture (MCAP), synchronized with ground reaction forces (GRF) and electromyography (EMG), to characterize the biomechanics and motor control of human bipedal posture has reached its zenith. To date, collecting data using these tools on healthy individuals (young and aging) and analyzing the metrics using statistical methods capable of dissecting the complex, non-linear behavior of biological signals has provided us with the following two things: (1) a fundamental understanding of postural control and (2) tools that can be used in the diagnosis, prognosis, and rehabilitative outcomes of individuals with disease, e.g., diabetic neuropathy, post-concussion syndrome, sports-related injuries, etc. Although these earlier methods had limitations in what their results could explain, past work segued, over the last two decades, into more advanced methods of data analysis, that is, machine (or deep) learning (ML) and artificial intelligence (AI). Using two general approaches, i.e., predictive modeling and data mining, ML and AI offer tremendous promise for advancing human movement research, improving medical decision-making, and enhancing rehabilitation programs [106,107,108,109]. Recent examples of the application of AI/ML include (1) augmenting the determination of joint kinematics and kinetics generated from marker and markerless MOCAP and synthetic biomechanical data [110,111], (2) the use of wearable technologies to better characterize walking and running biomechanics [112,113], (3) advancing the use of EMG for improving the diagnosis and classification of neuromuscular disorders, e.g., myopathies, amyotrophic lateral sclerosis, etc., [114,115,116], (4) improving the fidelity of models used to study postural control [117], and (5) adding to the existing knowledge of the measurement and control of bipedal human balance in both normal and aging individuals [118,119,120,121,122,123,124]. Our work examining the inter-muscular coherence of lower extremity muscles and muscle synergy’s role in postural control could be expanded by applying ML and AI methods.

Our study was not without methodological limitations. Future studies will need to include a larger sample size and test individuals with various other conditions, e.g., aging and musculoskeletal and neurological impairments. Unfortunately, because the raw EMG signals in this study were filtered, we were unable to examine coherence in the lower frequency band ranges, so future work will need to re-examine this issue. On the other hand, we believe the fidelity of analysis of the other frequency band ranges was preserved. We did not examine our EMG for cross-talk because our protocol was identical to Ojha et al.’s [86]; perhaps this assumption needs to be examined. Winter [41] suggested that the ankle invertor/evertor muscles were critical in the control of medio-lateral postural sway, yet we did not test the activity of any ankle evertors, e.g., peroneus longus. Future research using our protocol should include ankle invertor/evertor muscles. We defined leg dominance as the leg one would kick a ball with, yet there is no consensus on the definition of leg dominance, as some have suggested that the dominant leg is the one used to stand on one leg. Finally, since our test conditions were not randomly organized, a learning effect may have biased our results.

## 5. Conclusions

We believe that the use of connectivity analysis provides insight into how the CNS functions to control the multi-segmented musculoskeletal system under a variety of quiet standing postures. We have contributed additional evidence that the CNS can implement a common neural drive to simultaneously synchronize and activate key ankle muscles needed to maintain postural stability during internal perturbations, e.g., tandem stance with eyes closed. Some of our data corroborated previously published works, yet our data also uniquely suggest that muscle coherence in the lower and upper gamma frequency ranges may be related to the control of tandem standing. Additionally, it appears that functional muscle synergies are more important than simply limb dominance, although our data corroborate earlier studies that showed that in tandem standing, greater muscle activity occurs in the rear leg. The coherence analysis in this study was unable to identify changes in antagonistic or bilateral homologous connectivity with increasing instability, suggesting that perhaps additional information theoretic measures, e.g., mutual information (MI), might be useful in future research. Finally, the inter-trial and participant variability appears to be consistent with the principle of abundance, suggesting that formal information theory analysis of these data may be warranted. Our study results pertaining to young healthy adults suggest that the clinical testing of tandem balance tasks might be a useful adjunct to the clinical measure of balance for individuals with musculoskeletal and neurological impairments. However, to improve the fidelity of the clinical application of our methods, particularly for longitudinal testing, future research will need to develop more sophisticated analytical tools to explore, and perhaps explain, the intra-individual variability as it relates to biological versus methodological influences (i.e., a particular concern with surface electromyographic measures).

## Figures and Tables

**Table 1:** Neural frequency bands and possible origins.

Wave	Frequency (Hz)	Origin	Task Manifestation
Delta	0.5–4	Unknown	Isometric contraction, slow movements
Theta	4–8	Unknown	Isometric contraction, slow movements
Alpha	8–13	Unknown	Isometric contraction, slow movements
Beta	13–30	Motor cortex	Submaximal voluntary contraction
Lower Gamma	30–60	Motor cortex	Voluntary contraction, slow movements
Upper Gamma	60–100	Brainstem	Eye movement (60–90 Hz), respiration

**Table 2:** Plantarflexor (P) and dorsiflexor (D) muscles of the lower legs.

Posterior Muscles	Anterior Muscles	Deep Anterior Muscles
Medial gastrocnemius (P)	Tibialis anterior (D)	Tibialis posterior (P)
Lateral gastrocnemius (P)	Fibularis longus (P)	Flexor digitorum longus (P)
Plantaris (P)	Extensor digitorum longus (D)	Flexor hallucis longus (P)
Soleus (P)	Fibularis brevis (P)	
	Extensor hallucis longus (D)	

**Table 3:** Quiet standing balance test conditions.

Balance Condition	Description
EOFT	Eyes Open, Feet Together
ECFT	Eyes Closed, Feet Together
EOTanDF	Eyes Open, Feet Tandem, Dominant Foot Forward
ECTanDF	Eyes Closed, Feet Tandem, Dominant Foot Forward
EOTanDB	Eyes Open, Feet Tandem, Dominant Foot Back
ECTanDB	Eyes Closed, Feet Tandem, Dominant Foot Forward

**Table 4:** Neural frequency bands of interest.

Bands	Range (Hz)
Delta	0–4
Theta	4–8
Alpha	8–13
Beta	13–30
Lower Gamma	30–60
Upper Gamma	60–100

**Table 5:** Muscle pairs analyzed.

Left Unilateral	Right Unilateral	Bilateral Homologous
LTA:LMG	RTA:RMG	LTA:RTA
LTA:LS	RTA:RS	LMG:RMG
LMG:LS	RMG:RS	LS:RS

**Figure A1:**
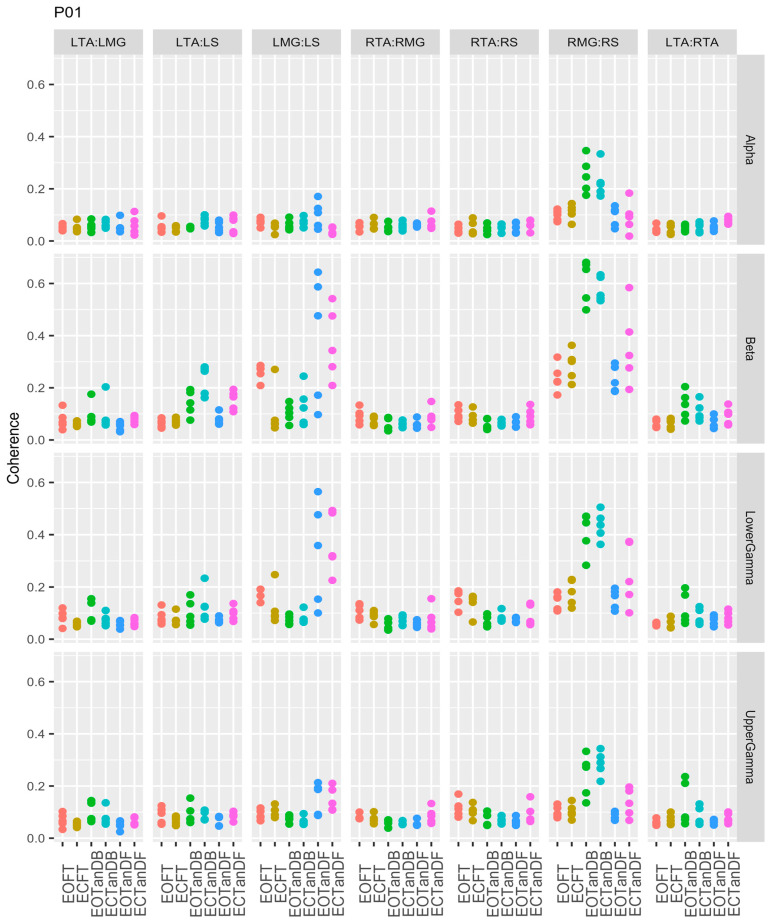
EMG-EMG muscular coherence for participant #1 (P01).

**Figure A2:**
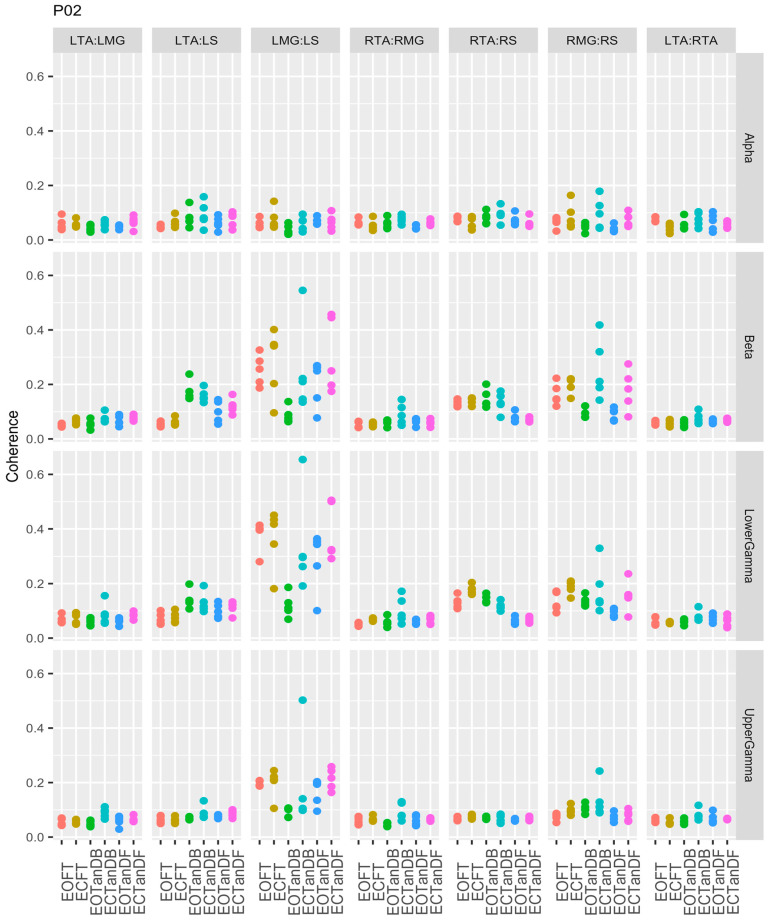
EMG-EMG muscular coherence for participant #2 (P02).

**Figure A3:**
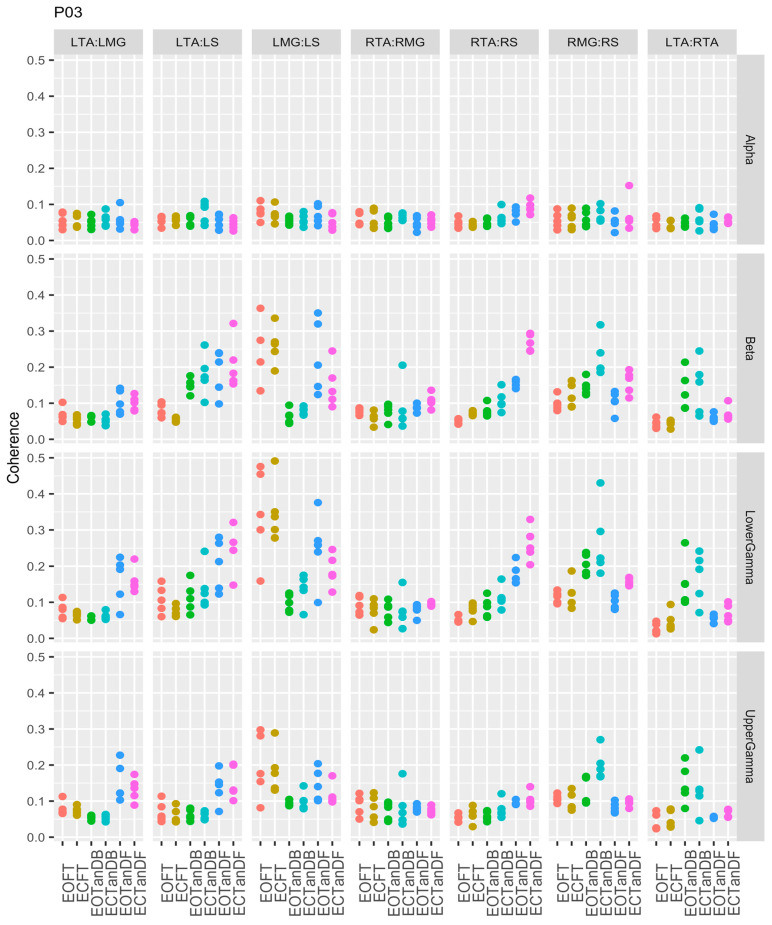
EMG-EMG muscular coherence for participant #3 (P03).

**Figure A4:**
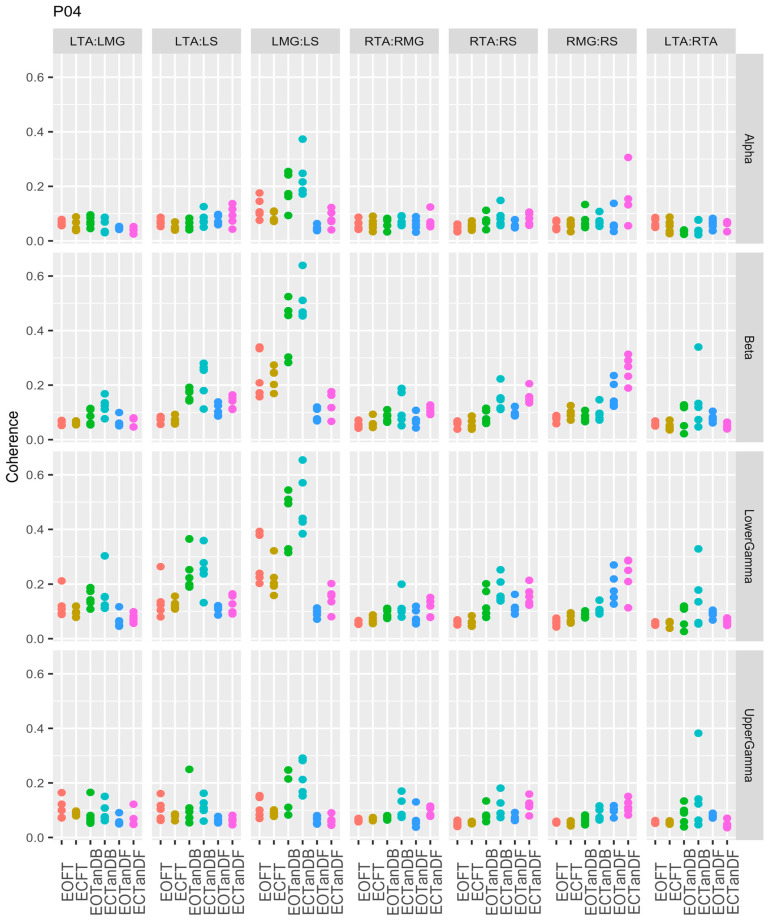
EMG-EMG muscular coherence for participant #4 (P04).

**Figure A5:**
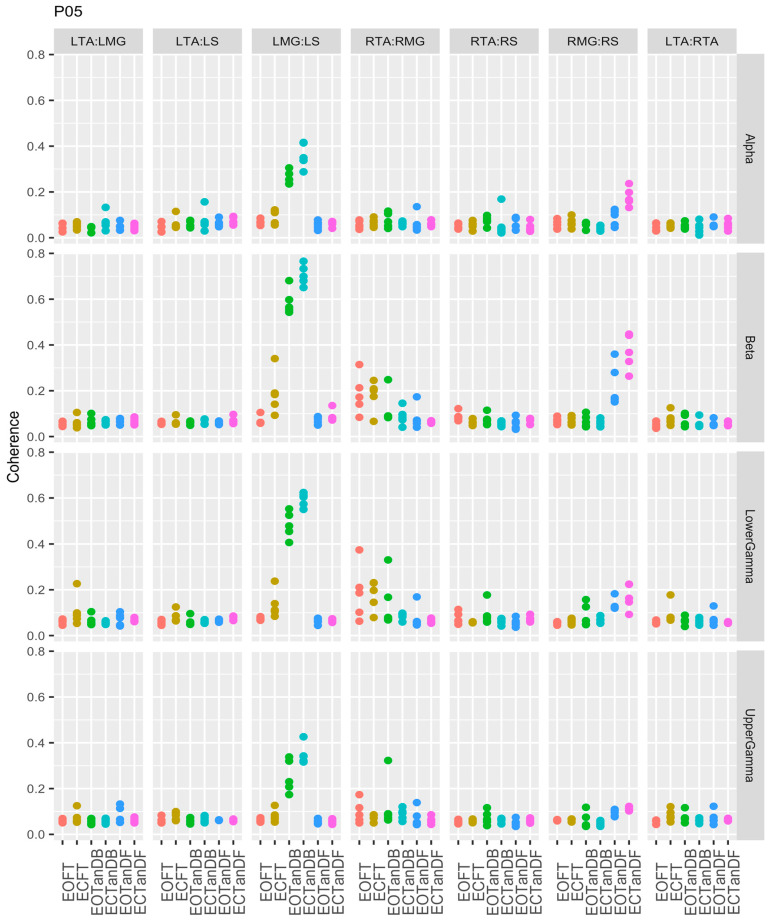
EMG-EMG muscular coherence for participant #5 (P05).

**Figure A6:**
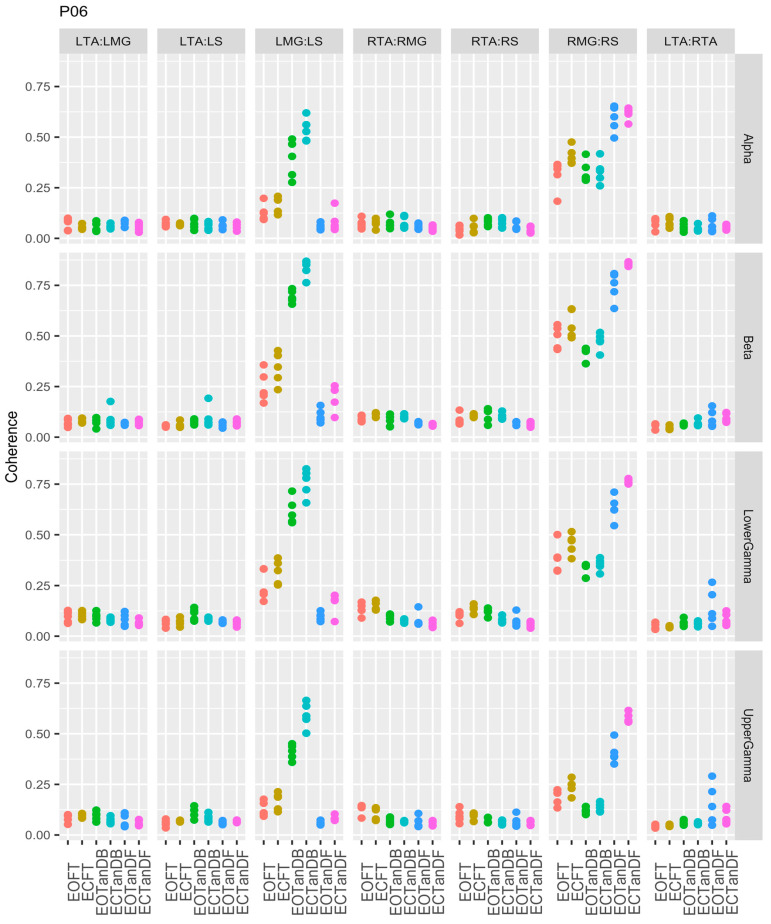
EMG-EMG muscular coherence for participant #6 (P06).

## Data Availability

The data presented in this study are available on request from the corresponding author due to restrictions imposed by our institution on retired faculty.

## Appendix A

EMG-EMG muscular coherence for each participant: five trials, over six test conditions (EOFT = eyes open feet together; ECFT = eyes closed feet together; EOTanDB = eyes open tandem dominant leg back; ECTanDB = eyes closed tandem dominant leg back; EOTanDF = eyes open tandem dominant leg forward; ECTanDF = eyes closed tandem dominant leg forward) for all muscle pair combinations (LMG/RMG = left/right medial gastrocnemius; LS/RS = left/right soleus; LTA/RTA = left/right tibialis anterior), and across four frequency bands: 8–13 Hz (alpha), 13–30 Hz (beta), 30–60 Hz (lower gamma), 60–100 Hz (upper gamma).
